# Experiences in running a complex electronic data capture system using mobile phones in a large-scale population trial in southern Nepal

**DOI:** 10.1080/16549716.2017.1330858

**Published:** 2017-06-14

**Authors:** Sarah Style, B. James Beard, Helen Harris-Fry, Aman Sengupta, Sonali Jha, Bhim P. Shrestha, Anjana Rai, Vikas Paudel, Meelan Thondoo, Anni-Maria Pulkki-Brannstrom, Jolene Skordis-Worrall, Dharma S. Manandhar, Anthony Costello, Naomi M. Saville

**Affiliations:** ^a^ Institute for Global Health, University College London, London, UK; ^b^ Mother and Infant Research Activities (MIRA), Kathmandu, Nepal

**Keywords:** mHealth, mobile data, electronic data capture, CommCare, ODK Collect

## Abstract

The increasing availability and capabilities of mobile phones make them a feasible means of data collection. Electronic Data Capture (EDC) systems have been used widely for public health monitoring and surveillance activities, but documentation of their use in complicated research studies requiring multiple systems is limited. This paper shares our experiences of designing and implementing a complex multi-component EDC system for a community-based four-armed cluster-Randomised Controlled Trial in the rural plains of Nepal, to help other researchers planning to use EDC for complex studies in low-income settings. We designed and implemented three interrelated mobile phone data collection systems to enrol and follow-up pregnant women (trial participants), and to support the implementation of trial interventions (women’s groups, food and cash transfers). 720 field staff used basic phones to send simple coded text messages, 539 women’s group facilitators used Android smartphones with Open Data Kit Collect, and 112 Interviewers, Coordinators and Supervisors used smartphones with CommCare. Barcoded photo ID cards encoded with participant information were generated for each enrolled woman. Automated systems were developed to download, recode and merge data for nearly real-time access by researchers. The systems were successfully rolled out and used by 1371 staff. A total of 25,089 pregnant women were enrolled, and 17,839 follow-up forms completed. Women’s group facilitators recorded 5717 women’s groups and the distribution of 14,647 food and 13,482 cash transfers. Using EDC sped up data collection and processing, although time needed for programming and set-up delayed the study inception. EDC using three interlinked mobile data management systems (FrontlineSMS, ODK and CommCare) was a feasible and effective method of data capture in a complex large-scale trial in the plains of Nepal. Despite challenges including prolonged set-up times, the systems met multiple data collection needs for users with varying levels of literacy and experience.

## Background

The increasing computing power, affordability and availability of mobile phones have made their use feasible and effective as an alternative to paper questionnaires in low-income settings. We reviewed literature describing experiences with developing and implementing Electronic Data Capture (EDC) systems in low- and middle-income countries (LMICs) in the past 10 years (search strategy available in Supplemental Appendix 1) and found that mobile devices are widely used in LMICs for healthcare delivery (mHealth) [1–8] including disease surveillance and control [9–15], epidemiological surveys [16–21], other research studies [22–24], remote data collection and monitoring [25–28] and public planning and mapping [29].

Evidence from systematic reviews and other sources indicate that EDC systems compare favourably with paper-based systems. EDC generally reduce data collection time [1,11,20]; improve data quality and completeness [1,2,5–7,11,20,23,30], largely through automated skip patterns, range and consistency checks in questionnaires; reduce data cleaning time [31,32]; reduce transmission delays and error rates [3,6,7,10]; and make reporting easier, faster and more reliable [10]. Mobile devices facilitated monitoring and supervision of health workers [3] and were cheaper than using paper [2,3,6,12,17,19,22,30,31].

The devices and software used varied according to the needs of the system being implemented and the technology available at the time. For example, basic phones transmitting coded SMS messages using software such as FrontlineSMS were used for simple information or event notification [33–35], and transmission of Global Positioning System (GPS) coordinates was used for mapping [27,35]. Smartphones (or handheld computers) were used to collect more complex data [4,14,15]. Mobile phone questionnaire software used included open-source or free-to-use options such as Open Data Kit collect (ODK) [4,15,26,27], Epicollect [15,27], EpiInfo [21] and CommCare [2,3,28], or commercial software such as Pendragon Forms [16,17,25] and Questionnaire Development System [22]. Others programmed custom-made systems [4,11,30,31].

Several papers have described experiences of using EDC, but most papers cited so far are for mHealth interventions rather than for research studies, and few share successful experiences from South Asia or from complex studies or cluster-Randomised Controlled Trials (RCTs) requiring longitudinal follow-up. The aim of this paper, designed to complement the trial protocol and main results paper, is to share our experiences in the design and implementation of an innovative multi-component EDC system developed for the Low Birth Weight South Asia Trial (LBWSAT) in Nepal, to enable other researchers in low-income settings to learn from and build upon them.

The RCT and its methods are described in detail in the trial protocol [36] and the impact in the main trial results paper (under review) so are not discussed in detail here. In brief, this large-scale community-based cluster RCT tested the impact of three interventions delivered to pregnant women on birthweight and child nutrition in the first 16 months of life. The interventions were: women’s groups using participatory learning and action (PLA), supplemented by limited home visiting of women who were unable to attend groups, focused on nutrition in pregnancy; PLA plus a monthly cash transfer; and PLA plus a monthly fortified food supplement; compared with a control arm receiving current government programmes. Eighty Village Development Community (VDC) clusters (20 per arm) were randomised, covering a population of 500,000, 64,000 reproductive age-women and 25,000 women who became pregnant and were enrolled and followed-up.

### Ethics

The trial was approved by the Nepal Health Research Council (108/2012) and the UCL Ethical Review Committee (4198/001), London, UK.

### Setting

LBWSAT was implemented in the plains (*Terai*) of Nepal, in Dhanusha and Mahottari districts where there is a combined population of 1.5 million [37]. The area is flood-prone with high humidity and temperatures. Travel over the large geographic area can be challenging due to poor-quality roads and poor public transport, especially during the monsoon. Mobile phone network coverage is reasonably good and improving. Literacy levels are low at 49%, and only 5% of women aged 15–49 have completed primary education [38]. However, at least 75% of households own a mobile phone, suggesting that mobile phones would be a feasible method for local staff to collect data in this context.

## EDC system design

### Data capture requirement of the LBWSAT

We needed ‘surveillance’ activities to monitor women’s menstruation, enrol them if pregnant, and follow pregnancies, births, and infant and young child outcomes. We also needed to monitor delivery of the interventions – the food and cash transfers, attendance of women’s groups and receipt of home visits.

Our key requirements were: real-time or quick access to data, automatically generated identification (ID) cards, links to data from previous questionnaires, multiple language options, and collection of GPS coordinates. We needed real-time data on pregnancies and births, so paper-based data collection was not feasible. Use of text messaging would allow interviewers to be informed immediately after a pregnancy or birth was identified, making it more feasible to weigh babies within 72 hours after birth. Quick access to data, by avoiding the need to enter data from thousands of paper forms, could also facilitate reporting of trial results in a timely manner. We wanted to automatically generate ID numbers as we had previous experience of fieldworkers making frequent errors when assigning and recording participant IDs. Use of photo ID cards to record food and cash transfers could also discourage casual fraud. Multiple interactions with each woman required access to previously collected data as interviews were being conducted, to control question routing. We needed the questionnaires to be available in three languages (Nepali, Maithili and English), which is easier to achieve with EDC than with paper. Lastly, we needed to monitor data collector performance and map the spatial coverage of trial participation, and use of phones would enable the collection of GPS coordinates.

**Figure 1.**
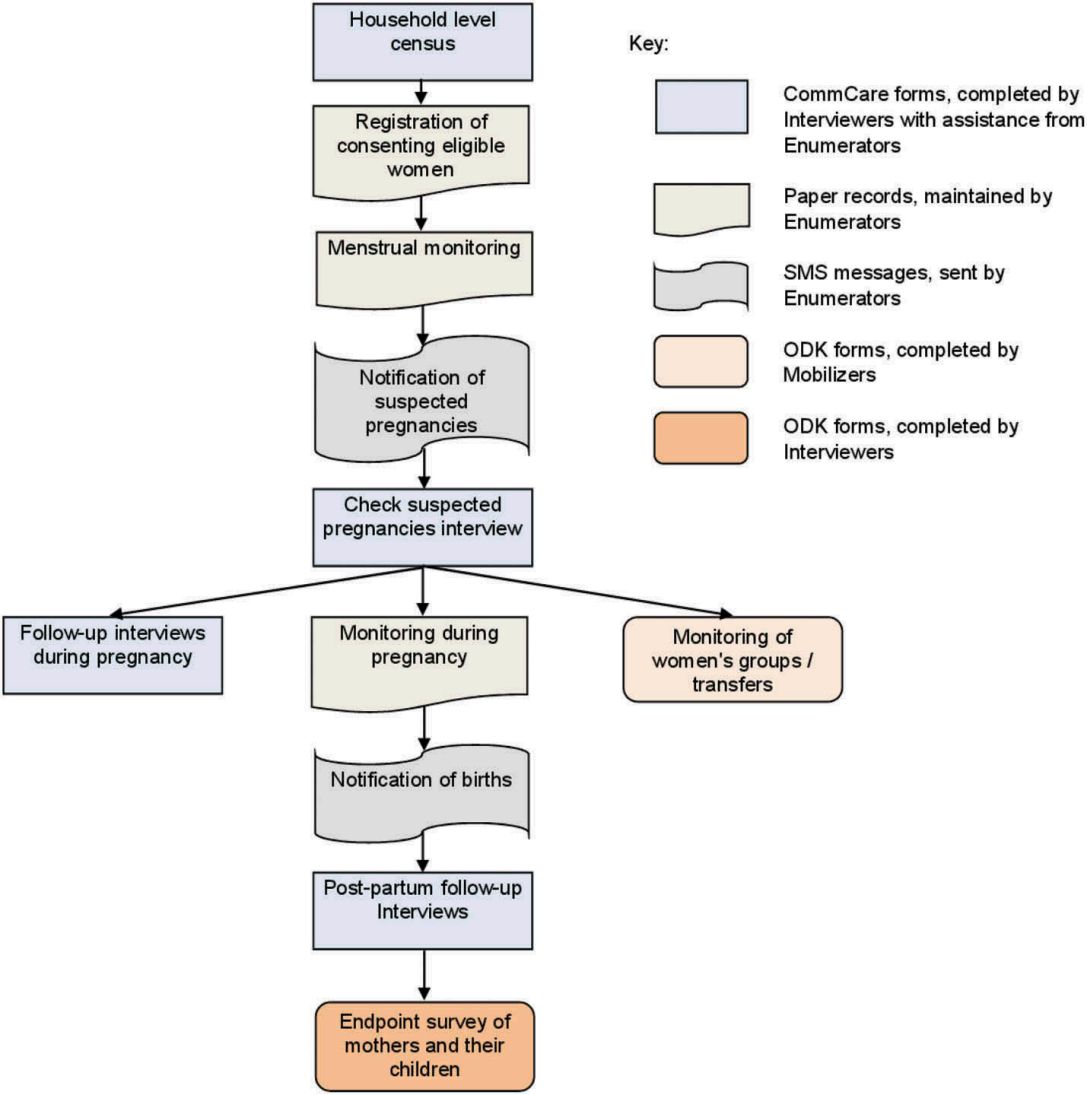
Diagrammatic representation of women’s interactions with the data collection system showing the different cadres and technologies used.

The data system was designed, implemented and managed by a part-time UCL advisor (BJB) and two MIRA data managers (AS, SJ), with inputs from other team members (NMS, SS, HHF, AR). The trial’s multiple data needs could not be met economically by a single EDC system (Figure 1, Table 1).

**Table 1.** Mobile devices used for LBWSAT surveillance.

Name of data collection cadre	Minimum years of education	Number employed	Roles	Data collection type	Technology used	Reason for choice of technology	Software /platform	Reasons for data collection type and method	Challenges experienced
Enumerator	5	720	Monitoring of mentruation, pregnancies, births, deaths and migration. Assisting data collectors with pregnancy testing and anthropomtery	2880 paper registers (4 per Enumerator) + automated text messaging	Nokia 1280 Ultra-basic phone c. $11	Very low cost. Ability to display Devanagari characters	FrontlineSMS to send simple coded text messages	Too expensive, and difficult to train and supervise all 720 Enumerators with smartphone technology. Automated text messages simple and low cost	Registers proved expensive to print and hard to transport /distribute due to large numbers. A few registers lost/damaged during use. Mobile phone network not available in a few places. Simpler register required
Interviewer	10	66	Enrolling and following-up women using questionnaires in early and late pregnancy, within 72 hours of birth, 42 to 300 days after delivery and at trial endpoint when children were 0–24 months old	Electronic questionnaires	Samsung Galaxy Y Android smartphone c. $160	Lowest-cost Android phone from a well-known brand available	Collect data with CommCare for trial surveillance or ODK for endpoint survey	CommCare allowed case management system where each data collector was allocated cases to follow. These displayed only on allocated data collectors’ phones. ODK had no user fees so cheaper to user for endpoint survey without case management but utilised data collected during the previous surveillance	Small number of cases of lost data which had to be collected again due to system error. Mobile phone network not available in a few places. Limited breakages/damage but much less than lower-cost phones. Few lost or stolen
Supervisor	12	16	Supervising Enumerators and Supervisors. Collecting duplicate readings. Collecting ‘double-entered’ questionnaires while Interviewer asked questions. Filling observation checklists of data collector performance	Electronic questionnaires					
Senior staff	14	4	Supervising all data collectors, Supervisors and Coordinators. Filling checklists whilst observing interviews or women’s groups	Electronic questionnaires					
Mobiliser	5	540	Scanning participants’ QR codes on ID cards and filling basic questionnaires tracking intervention delivery including: food and cash transfer delivery; women’s group participation; home visits to pregnant women	Pictorial electronic questionnaires	Karbonn A1+ Android smartphone c. $60	Lowest-cost Android phone available	Collect data with ODK Collect	Too expensive to provide higher-cost (branded) smartphone so lower-cost models utilised. ODK used due to lack of user fees and because simpler forms were needed than for trial surveillance	Lower-cost smartphones had poor memory and were more prone to breaking. Husbands of Mobilisers took phones for own use and wiped memory. Small number lost /stolen. Mobilisers found the phone difficult to use
Women’s Group Coordinator	10	30	Supervising Mobilisers. Filling checklists whilst observing groups	Electronic questionnaires

Therefore, our system was built around three free-to-use software packages: FrontlineSMS, ODK and CommCare. Reliable IDs presented as QR codes on participant ID cards could be scanned with smartphones, simplifying the process of linking data from different sources. Operational reasons for selecting the hardware and software used are discussed in Table 1 and Supplemental Appendix 2. Six cadres of salaried staff and incentivised volunteers used the system. Their roles, data capture systems employed, and the reasons for choosing them are outlined in Table 1.

### Formative testing

During trial inception, formative data and a population census were collected using CommCare on Samsung Galaxy Y Android phones. This demonstrated that literate data collectors could operate the phones and software with ease. Unfortunately, there was insufficient time to thoroughly pre-test the use of low-specification Android phones and ultra-basic phones with lower-literacy users (Mobilisers and Enumerators, respectively).

### Registers for menstrual monitoring by Enumerators

**Figure 2.**
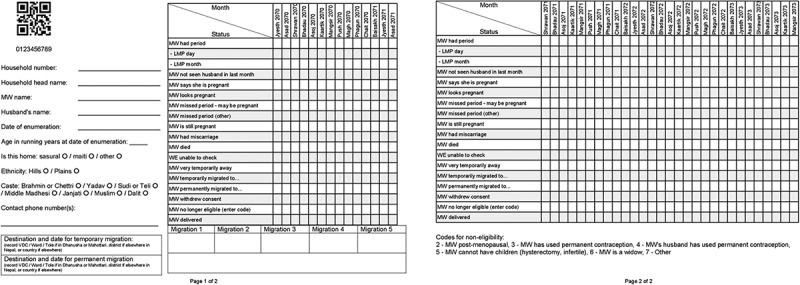
Example register pages for one woman (English version).

We needed a fail-safe system of tracking women from menstrual monitoring (for pregnancy detection) to follow-up of pregnancy, birth and child growth. We designed a paper register for Enumerators to track menstruation and pregnancies of women in their working areas. Each page of the register was pre-printed with a unique woman ID number, both as plain text and as a QR code. These IDs encoded the women’s study arm allocation and cluster, to facilitate appropriate skip patterns in the forms, and to help ensure that women from non-transfer arms did not receive transfers (see example register in Figure 2).

We identified 63,308 eligible married women of reproductive age (see the trial protocol for eligibility criteria [36]) via a household census between August and November 2013. The names and contact details of each eligible woman were recorded in the paper registers, so every enumerated woman had her own unique identifier from the point she entered menstrual monitoring. After the initial census, we prospectively added women to the registers when they moved into clusters. Seven-hundred and twenty incentivised volunteer Enumerators used these registers to monitor and record the menstrual status of every eligible consenting woman in their ward each month, together with other significant events including pregnancy, miscarriage, delivery or death.

We provided Enumerators with ultra-basic phones to notify the system of significant events by sending a simple coded one-character text message to a server, which was running the FrontlineSMS text message handling system (Supplemental Appendix 1). FrontlineSMS then sent an SMS to the relevant Interviewer requesting action (for example, collect baby weight). Messages received and sent were logged to monitor Interviewer and Enumerator performance.

### Enrolment and follow-up by interviewers using CommCare

On receiving notification of a suspected new pregnancy or a birth, the Interviewer called the Enumerator to confirm the event had taken place and arrange a time to meet. In the case of a newly identified pregnancy the Interviewer and Enumerator visited the woman to conduct a ‘pregnancy confirmation’ interview, after which up to five follow-up interviews were conducted between pregnancy verification and 42 days after delivery, plus an endpoint questionnaire. In the case of a delivery, the Interviewer was required to visit the household to weigh the baby and conduct an interview within 72 hours of birth. A vital status recording form was also created for Interviewers to capture vital event and migration information from the Enumerators’ registers every month.

Interviews were administered using the CommCare data collection platform, on medium-specification Android smartphones (Table 1). A key reason for using CommCare as the main data collection platform for follow-up was its ‘case’ function. Mobile phone data collection software typically treats each form filled as an independent entity: the data are collected, then sent to a server and removed from the phone. CommCare, however, can store a sub-set of form data more permanently on the phone, and use (and update) that information in subsequent interactions with the same person, which was very useful in our context. We used two case types – ‘Woman’ and ‘Pregnancy’. The Woman case kept track of enrolled women’s pregnancy status (pregnant or not) and the number of pregnancies the woman had in the study. The Pregnancy case kept track of numerous variables associated with the pregnancy, such as the estimated delivery date (EDD) and which questionnaires had been administered. We used case information to make relevant forms available to Interviewers at different gestational ages. CommCare case data may either be shared amongst all Interviewers, or restricted to the original collector. We chose to do the latter, but could transfer cases to other Interviewers as necessary.

### Photo ID cards

**Figure 3.**
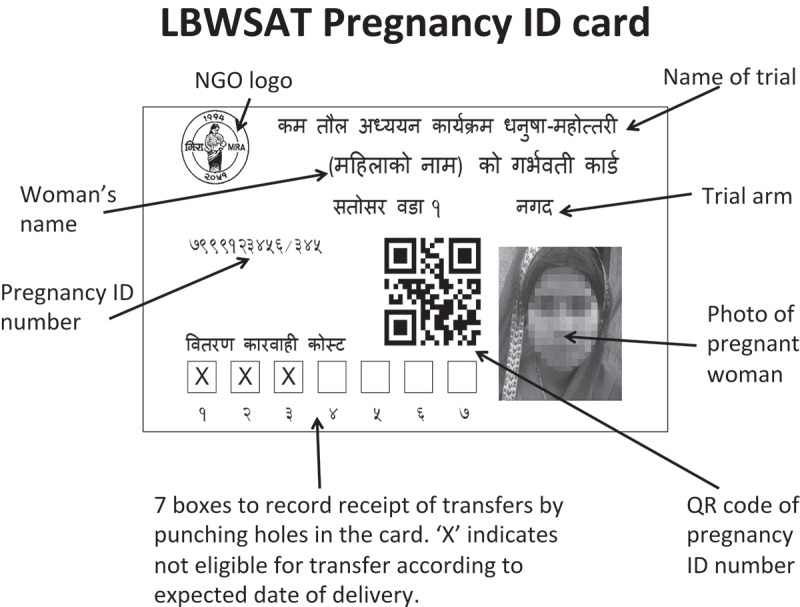
Example ID card (actual size 85 × 54 mm).

When a pregnancy was confirmed, the CommCare form automatically generated a Pregnancy ID, based on the woman’s Woman ID, her pregnancy number (i.e. how many times she had registered a pregnancy within the study) and an encoded version of her last menstrual period date. Upon consent, the Interviewer photographed the pregnant woman, the photograph being used to make a barcoded photo ID card that every pregnant woman received after enrolment (Figure 3). We developed a report in Microsoft Access to lay-out the credit card-sized ID cards and printed them in monochrome (to minimise costs) on Datacard SP30 card printers. Interviewers checked the names and photos at the start of each interview and scanned the barcode on the ID card, which gave access to the case data. ID cards helped ensure that transfers went to the right women as ID cards were checked against the person receiving the transfer.

### Food and cash transfer system and ODK

Five-hundred and thirty-nine Mobilisers tracked the delivery of food and cash transfers to enrolled pregnant women using forms developed on ODK and administered on low-specification Android smartphones. ODK, an open-source toolkit for development of data collection on mobiles, was selected primarily because it is free to use, whilst having all the features needed. These included a server component (ODK Aggregate), easy management and use of multiple languages in one form, automated skip patterns, ability to display pictorial images associated with questions or interview sections, QR code scanning, GPS recording, and caching of data on the phone until synchronising was possible. Cost was a significant factor due to the high number of Mobilisers using the system.

Mobilisers recorded the monthly delivery of food and cash transfers using two parallel mechanisms. For accounting purposes, we collected recipients’ signatures or thumb-prints on printed lists with ID numbers and names. For electronic tracking of transfer delivery, we scanned QR codes from pregnancy ID cards. Seven boxes on each ID card indicated the maximum number of transfers for which the woman was likely to be eligible, based upon gestational age at enrolment and the EDD. A hole was punched in each box when a transfer was delivered. If a woman enrolled late in her pregnancy, a cross was printed inside a box to indicate that the woman was unlikely to be eligible for that transfer (Figure 3). We also used ODK to collect endpoint data cross-sectionally on each woman–child dyad at the end of the trial, to capture anthropometric and other outcomes on children 0–24 months in age (average 10 months).

### Electronic forms development

We designed ODK forms by creating an Excel workbook that described the form. This facilitated the sharing of form designs at the development and piloting stage, and provided a useful overview of the questionnaire content and structure. We used a free online service to convert Excel to .xml files before uploading the questionnaires on phones (http://opendatakit.org/xiframe/). Although it was possible to create some forms for CommCare in a similar way, forms that used the case data feature (as nearly all of ours did) needed to be designed using a web browser interface to the CommCare server, which necessitated a reliable and reasonably fast Internet connection.

### Data transfer and processing

Data from CommCare interviews were transferred to the CommCare server using the mobile phone network or office WiFi. Interviewers were asked to connect their phones to the Internet to send data daily to guard against data loss. Similarly, when an Internet connection was available, data collected with ODK were transferred to a server running the ODK Aggregate software, using a Google App Engine (AppSpot). For the few Mobilisers unable to access adequate network coverage, data were transferred to a laptop computer using a USB connection by team members visiting the cluster on motorcycles.

In the field office we installed as our local server a high-specification laptop, with mirrored hard disks, from a reputable manufacturer (Dell). We did not use a conventional server system because of intermittent electricity supply in the office and the probable need to provide such a system with air conditioning. FrontlineSMS ran on this server using an Internet dongle to send and receive text messages.

On our server, we developed and ran two automated downloading systems, one for CommCare and one for ODK. Because of the volume of data collected and the multiplicity of different data collection instruments, we did not want to manually download data-sets, which is the default way of accessing CommCare and ODK data. We used Stata 13.1 to generate appropriate cURL (‘see URL’) commands to interact with CommCare’s web Application Programming Interface (API), a paid-for feature, to download the CommCare data. Similarly, we used Stata to generate commands for ODK Briefcase (a program designed to manage ODK data on a local computer) to download the ODK data. Both the CommCare API and ODK Briefcase support incremental downloads: CommCare by passing a unique token obtained from a previous download back to the CommCare server, and ODK Briefcase by it checking that records have not previously been downloaded. Both systems created data on our server as .csv files, converted them to Stata format, created variable and value labels, and generated indicator variables for questions that allowed multiple answers. Because, at the time, Stata did not understand Unicode characters in text variables, we transliterated UTF-8 Devanagri text into Latin script using a Mata subroutine within Stata. For the ODK data, we used the user-written Stata add-on odkmeta (Matthew White, Innovations for Poverty Action, New Haven, CT) to automatically assign variable and value labels to variables from the Excel spreadsheet definition of the form. For the CommCare data, we manually used the ‘Export Form Contents’ feature of the web interface to create a local Excel file describing the form design, which we read in Stata to generate the necessary labelling and recoding commands. Stata versions of the data were automatically stored on Dropbox, allowing authorised researchers access to near real-time data from anywhere in the world. Although we could easily have made these systems completely automatic, we chose to manually run them every morning to ensure that any errors or issues would be more easily identified. Data files were merged in Stata using participants’ unique IDs.

### Monitoring and supervision

Supervisors, Women’s Group Coordinators, and senior staff monitored performance in the field. Sixteen Supervisors monitored four or five Interviewers each using observation checklists and duplicate data entry forms developed on CommCare. Each of 30 Women’s Group Coordinators observed and supported on average 12 out of the 18 groups they were each responsible for every month. For each meeting observed, the Coordinators assisted Mobilisers with QR code scanning when delivering transfers, recording women’s group attendance and keeping paper accounting records. Coordinators also completed women’s group observation checklists using CommCare forms, as part of process monitoring.

### Data quality checks and user performance reports

We used CommCare’s inbuilt report functionality to show the number of forms filled by each interviewer per day or month. We also prospectively tracked follow-up and interviewer performance using the merged data and Stata do files. From these we produced individual interviewer performance monitoring reports to enable Supervisors to track progress and facilitate conversations with underperforming Interviewers. Performance indicators included time taken to perform interviews, digit preference in birth weight and length, proportion of records with a GPS location, total number of interviews completed in a day and in total, and scatter graphs for visual review of birth weight measurements over time. Average interview time was calculated using date and time stamps at the beginning and end of questionnaires.

## EDC system implementation

### Interviewer training on EDC

Data management team members trained Interviewers on use of phones over a series of sessions between July 2013 and June 2014. Interviewers and Supervisors received 24 and 28 days of training respectively on questionnaire content, anthropometric measurement and phone operation. Supervisors supported Interviewers to train the Enumerators for four days on use of ultra-basic phones, filling the paper registers and assisting with anthropometric measurements. Women’s Group Coordinators received 2–3 days’ training on how to operate phones and collect observation checklist data as well as how to use the phones used by Mobilisers. They then trained Mobilisers in groups of 18 for 2 days each on use of smartphones and ODK forms and provided on-going training and support during women’s groups and food/cash transfers.

### Menstrual monitoring, main surveillance and intervention tracking

Our mobile phone data collection systems were used in the field by 1371 staff between December 2013 and October 2015. Out of 25,089 enrolled pregnant women, 21,147 (84%) socio-economic questionnaires, 13,847 vital status recording forms and 17,839 follow-up forms were completed using CommCare. At endpoint, forms were filled for 19,222 children and 19,017 mothers using ODK. In terms of intervention tracking, phones recorded 47% of the 28,527 cash transfers and 57% of the 25,675 food transfers provided using ODK. Of around 10,070 meetings held over 2 years across the 539 women’s groups, 5717 group attendance forms and 5594 meeting observation checklists were filled on mobile phones (covering over 50% of meetings). The average interview time for the delivery, post-neonatal, early and late pregnancy questionnaires was 12.6, 22.9, 22.3 and 30.8 minutes, respectively. An average of 63% of GPS positions were recorded, ranging from 50% to 70% across the six main questionnaires.

## Experiences in running EDC systems

### Advantages of EDC systems

Once the data collection tools were set up, EDC sped up a number of processes around data collection, storage and data processing. Time was saved on database building, data management, data entry and backup, as well as for data cleaning and recoding because this was largely automated via Stata script files. Creation of photo ID cards for more than 25,000 women, with participant ID numbers embedded in barcodes, successfully facilitated prospective follow-up by Interviewers and helped ensure correct identification of participants. As each record in the downloaded data had the pregnancy ID number of the woman reliably attached to it (scanned from the participants’ ID card), we easily merged information using this identifier. Use of FrontlineSMS was effective in facilitating communication between Enumerators and Interviewers. However, timely birth weight measurements were challenging to achieve for a number of reasons that were not specific to the mobile phones, including late detection of deliveries by Enumerators and gaps between Interviewers receiving a message and responding to it. Fifty-three percent of birthweights were measured within 72 hours and 68% within 10 days of delivery. Forms with automated skip patterns and loops reduced interview times and the prevalence of missing values. This was particularly advantageous as some questionnaires took over 30 minutes to administer, over and above the time needed to take anthropometric measurements. The daily downloading and automatic processing of data facilitated the set-up of real-time, automated progress and quality checks on incoming data. This enabled researchers to easily identify poorly performing data collectors. The immediate availability of data also made it possible to generate automated follow-up lists, so interviewers knew whom to visit and when, although this happened later on in implementation. Barcode scanning added a level of ‘accountability’ to the transfer distribution, which helped reduce attempts to defraud the system. Although a challenge, we successful incorporated the Nepali calendar (Bikram Samvat – BS) onto the phones. BS was not supported by the Android operating system we used and BS dates are not algorithmically convertible to the Gregorian calendar. We used a combination of lookup tables (for very important dates, such as dates of birth) and approximation for less precise dates such as Last Menstrual Period (LMP) to get around this problem. Finally, CommCare’s case data function ensured that follow-up forms could only be filled once preceding forms had been completed which helped to ensure that crucial questionnaires (e.g. of birth-related data) were not missed.

### Challenges of EDC systems

We experienced a number of challenges, mostly related to the time and resources required to complete the complex study activities. Although the data system was designed around existing software, limited time and resources meant that the systems had to be developed as the study progressed, partly as a result of pressures to start fieldwork. All aspects of the system development took longer than we expected, especially the finalisation of the content of the follow-up questionnaires, and their testing and implementation in CommCare. We had no previous experience of using CommCare, so we paid for some on-site assistance from Dimagi, the creators of CommCare. Our CommCare inexperience, the length of some of the follow-up questionnaires, and complications such as the need to handle Nepali dates and to reference data from previous interactions in later CommCare forms all slowed us down. This meant we had insufficient time to check the usability of the ODK forms used by the Mobilisers. The large number of Mobilisers (540) also made it difficult for the small local data team to provide support. Planning for a longer set-up period and having more modest data collection aims would have reduced the total time needed for data management and enabled us to start fieldwork earlier.

Due to time pressures, it was not possible to develop all quality control procedures in advance of data collection. Although automatic skip patterns, plausible ranges and restrictions were added to the electronic forms, quality checking became much more efficient and thorough after developing the performance monitoring system. We had expected that CommCare reports could drive the process of following-up women; however, this functionality was not available so we implemented our own system later. More women enrolled than predicted, especially in transfer arms. We also overestimated the volume of data that could be collected from each participant and the feasibility of making five separate follow-up visits to each woman. It would have been better to streamline the data collected and make one visit in early pregnancy, followed by others in late pregnancy (possibly on a subsample), immediately after birth and finally after 42 days. If follow-up lists had been provided from the start, the volume of data to be collected per participant and the case-load per data collector reduced, and increased supervision provided, we might have improved follow-up rates and reaction times for measurement of birth weights, as we would have been less reliant on the Interviewers’ motivation and organisational skills.

Both Android phone models used were of modest specification, primarily chosen for budgetary rather than technical reasons. Their limited internal memory capacity and Central Processing Unit speed limited the size and complexity of the forms we could deploy and meant some of our larger data collection forms took several minutes to open. Battery life was also an issue, especially as the phones aged.

Electronic reporting of transfers distributed by Mobilisers was only around 50% of the transfers recorded using paper, which was lower than anticipated. Reasons for this included malfunction of low-cost smartphones, lack of network coverage, short battery life and lack of electricity to charge mobiles, and Mobilisers’ lack of skill in operating phones. Raw data included multiple form submissions for the same respondent or group on the same day because Mobilisers were unsure if the form had saved or not, so duplicates had to be excluded in subsequent processing. We believe that better-quality phones, reduction of information collected and simplification of the forms so that they were more manageable for Mobilisers, and additional piloting, training and support in using the phones and forms would have improved electronic recording of transfers. An inbuilt function to avoid multiple scans of the same QR code in one day, which would have been easy with CommCare, but was not with ODK, would have stopped duplicate submission.

It was important to ensure women’s vital status was recorded frequently to track deaths and miscarriages; however, this proved difficult for Enumerators to manage using the paper registers. Less educated Enumerators found the registers difficult to follow due to the large number of different rows that could be filled, and consequently some vital status information was not well recorded. Supervision of Enumerators by Interviewers who were overloaded was also insufficient. We planned to collect the status of each enrolled woman electronically every month from the Enumerators’ registers, using the vital status recording form, but this was not prioritised consistently by all Interviewers. A solution would have been to organise an endpoint census to establish the vital status and pregnancy outcome of all participants, but this was impossible due to insufficient resources.

A number of contextual challenges were also experienced. Although network coverage has improved in the *Terai* in recent years, some areas in the east of the study site still had no coverage. In those areas, ODK data collected by Mobilisers had to be uploaded to a laptop by a roving data assistant, rather than being sent to the cloud server. Additionally, although both CommCare and ODK support the distribution of revised form versions over the Internet, in practice all phones needed to be brought to the office for updates, which added time and cost, especially given the large geographical area and poor transport infrastructure. For CommCare, this was because we typically updated several forms together, and Internet updates would have taken an unacceptable time on the 2G networks in rural areas. Also, if we attempted to update phones without bringing them to the office, we would not know that all phones had been successfully updated, except by looking at the data subsequently collected. For ODK, we felt it would be too complicated for the Mobilisers to update their phones. The proportion of GPS points taken was low, and varied considerably between Interviewers. This was partly due to insufficient training in how to consistently get a GPS reading quickly, as well as staff impatience in doing so. The utility and/or social status associated with smartphones in the community sometimes hindered EDC, for example, there were cases where Mobilisers’ husbands ‘borrowed’ their phones, replaced the phones’ operating system and wiped the memory cards to use them for other purposes. The public electricity supply was very unreliable, especially in rural areas and in winter, so extra batteries or power banks were provided for Interviewers with particularly limited access to electricity.

Finally, Dimagi, who developed CommCare, changed the behaviour of CommCare at least twice during our fieldwork, such that we had to adjust our system to respond to the new behaviour. Setting up our own CommCare server could have alleviated these problems; however, we did not have the resources to do this.

## Discussion

During LBWSAT, 1371 staff members successfully enrolled 25,089 women and collected follow-up data using three mobile phone data collection systems – FrontlineSMS, ODK and CommCare – and a number of supporting software products. The concurrent use of multiple systems was feasible and effective for a complex large-scale trial collecting real-time longitudinal data from numerous different activities. The system responded well to varying end-user needs, and field locations with limited connectivity. It was possible to tailor the system to context- and project-specific needs, considering factors such as staff capacity, Internet availability and phone specifications. Whilst EDC has been used successfully in Nepal [20], to our knowledge, few experiences have been shared of EDC using multiple interlinking systems to facilitate a complex cluster-RCT in a South Asian setting. By demonstrating how mobile devices using open-source software can be integrated into a large automated data management system, this study adds to the growing literature documenting the feasibility of EDC in rural and resource-poor settings for research or epidemiological surveys [16–20,22,23,30,31].

Particular benefits of our system included increased efficiency in daily downloading through automated recoding and merging of data, real-time tracking and follow-up of cases using a unique ID system with QR codes, and tracking and automated quality checks of data collector performance. In a systematic review, Hall et al. [6] identified key issues common to implementing mHealth interventions in low-income settings, including identifying vital events on time, following-up informants and establishing computerised field data collection systems at scale. Others have experienced challenges with generating unique identifiers for tracking participants or merging data longitudinally, sometimes resulting in duplicates [4,22,39], or errors from the use of the same ID for multiple patients [4]. Our study was largely able to overcome these challenges by scanning QR codes on ID cards containing unique ID numbers at the time of data collection.

On the whole, data collectors with limited training were able to use the phones; however, this varied by end-user. Use of basic phones by low-literacy incentivised volunteers was successful as a means of alerting Interviewers to events requiring rapid response. Similarly, use of medium-specification smartphones by employees with at least higher secondary education resulted in good data quality. However, use of low-specification smartphones by incentivised volunteers (Mobilisers) with limited literacy skills was less successful due to a combination of hardware problems and challenges experienced in using the phones. Experiences from similar contexts indicate that with adequate training (generally around 2–3 days depending on the task), low-literacy users are able to successfully use mobile phones for data collection and send SMS messages [7,18,34], and those with more exposure to phones at home needed less training [34]. In their research projects in rural Malawi, King et al. [31] reported that although most data collectors did not have prior experience of EDC, this was not a barrier and one to two weeks’ training was sufficient. Perosky et al. [35] however found that the error rate in EDC was significantly higher for traditional midwives than for the more educated certified midwives, and that years of education was the only predictor of successful SMS transmission. Similarly, we found that Mobilisers with a minimum of 5 years of education found smartphones difficult to operate and the forms hard to fill without supervision compared with our more educated Interviewers/Supervisors. We believe our challenges would have been largely overcome with better-quality phones, simpler forms and more training and support provided to Mobilisers. This highlights that EDC systems must be tailored to the needs of the end-user. In low-income settings, some users (especially, in our case, community volunteers) have low literacy levels, so the types and complexity of data collection tools need to account for this.

The main limitation of this study was insufficient time and resources allotted to the set-up phase and too much data to be collected per participant and per data collector. While the trial benefited from the use of multiple freely available software and hardware products, initial system set-up time hindered progress at the start. It was not possible to develop all quality control and performance management systems in parallel with design and set-up of the system, which meant that tracking of participant follow-up and interviewer performance began later than intended. Inadequate set-up time has been frequently reported and Byass et al. [17] also reported delays in implementing quality control procedures as a result. Paudel et al. [20] highlight that sufficient time must be allocated to designing and pre-testing electronic questionnaires, particularly for questionnaires in multiple languages and in a non-Latin script. More preparation is needed for developing questionnaires and testing in EDC than for paper-based surveys [5]. However, this front-loaded effort considerably reduces the time needed for data checks, quality control of issues like skip sequences and data outside plausible ranges, data entry system set-up, data entry and cleaning [16]. DeRenzi et al. [2] recommend that several months of refinement should be scheduled after initial development of complex data systems using CommCare. These experiences highlight the need for set-up times to be adequately planned and budgeted. A run-in period is crucial to test the systems. Prior to data collection, time should be allocated for the design of performance monitoring procedures, to fully maximise the scope for EDC systems to improve data quality and follow-up rates.

The necessity of skilled EDC development capacity has been highlighted as a frequent challenge in EDC implementation [5,17,18], without which implementation can be slowed down, or the potential of EDC not fully maximised. This matches our experience in that we needed a wider range of skills than would have been needed for a paper-based system, and more data management time than we planned for.

In prospective studies, follow-up rates rely on data collector motivation and workload, so recruitment, training and effective supervision of sufficient data collectors, and realistic targets are key. Regularly generated lists of follow-ups required helped Interviewers manage their workload and enable researchers to better track progress. We planned to implement a workload management system for Interviewers within CommCare, but this was not possible. Due to time limitations, we were unable to develop follow-up lists until several months after data collection had started, which made workload management more difficult for Interviewers. Follow-up lists have been used successfully by others, for example, DeRenzi et al. [2] reported that follow-up lists helped community health workers to identify who needed further attention, and motivated them. Knipe et al. [23] reported that daily and weekly status reports allowed supervisors to identify missed households and monitor data quality. Such systems need to be implemented from the start to facilitate Interviewers planning their workloads.

We experienced a number of contextual challenges, similar to those reported elsewhere. Poor network coverage in a few of our clusters inhibited data transfer and back-up, an issue we and others overcame by storing data on SD cards until it could be downloaded directly onto laptops [11,22,26]. Network issues are a frequent problem with EDC implementation [2,5,10,12], sometimes resulting in duplicate data submissions for the same participant or case [2,10,14,29], or infrequent data submission [13]. In our case, duplicate entries were easily removed in Stata. We struggled with short battery life and found that charging some devices could be challenging due to frequent power cuts. We therefore provided chargeable ‘power packs’ that could be charged when electricity was available, alongside phones. Successful solutions used by others include use of solar chargers [4,16,17,25,26], motorcycle batteries [17] and spare batteries [39]. Like others, we found strategies for saving battery life included minimising use of GPS, Bluetooth and WiFi [4,39] and using phones with longer battery life. Such contextual constraints should be considered in advance to minimise future problems.

In contexts where different language scripts and calendars are used, such as in Nepal and Ethiopia, systems may need to be customised [4]. Although Little et al. [4] found errors in the calculation of expected date of delivery were created when users changed English dates to match the Ethiopian calendar [4,22], we avoided such problems by allowing interviewers to enter Nepali dates, converting these to English calendar dates using Stata and approximating Gregorian calendar dates within the CommCare forms. We also locked users’ access to changing settings on phones, which helped to avoid deletion of necessary data and applications, downloading of large files and installation of apps that might disrupt data collection.

## Conclusion and recommendations

Our experience suggests that implementing EDC systems in complex research studies with multiple data sources across wide geographical areas is possible. Essential prerequisites include: sufficient set-up times and expertise for design, roll-out and management, devices with sufficient specification, and awareness of contextual issues. A fail-safe participant identification system and tailoring to end-user needs are crucial. Sufficient training and ongoing support must be provided particularly for low-literacy users or resources must be allocated to employ more educated users.

## Supplementary Material

Supplemental Data

## Paper context

A literature review on experiences of electronic data capture in low-income countries revealed limited information from complex research in South Asia. Our experience implementing a multi-component electronic data capture system using mobile phones showed that linking three open-source platforms to capture multiple data sources was feasible in Nepal. Using a reliable identification system, we successfully followed-up > 25,000 cluster-Randomised Controlled Trial participants. Sufficient time/expertise for set-up and support for low-literacy users are crucial.
