# Supplementary material for: Experiences in running a complex electronic data capture system using mobile phones in a large-scale population trial in southern Nepal
Source: Glob Health Action. 2017 Jun 14;10(1):1330858. doi: 10.1080/16549716.2017.1330858 (PMC5496067; doi:10.1080/16549716.2017.1330858)
Supplement: Supplemental Data [file zgha_a_1330858_sm5370.docx]

**Supplemental online material**

**Web appendix 1 – Literature search strategy**

We searched for peer-reviewed papers published between 2007 and 2017 that reported experiences of using electronic data capture in low and middle-income countries (LMICs). We searched Pubmed/Medline and Web of Science, using the search terms or medical subject headings provided below, using ‘OR’ to join words within columns, and ‘AND’ to join groups between columns.

This resulted in 639 references, after deleting 31 duplicates. We limited our review to relevant, satisfactory quality studies that included any reference to researchers’ or enumerator’s experiences of using mobile phones, computers, tablets or any other handheld data entry device to collect data, including surveys, on-going surveillance, or monitoring data. Four authors filtered the results to exclude any papers not on experiences using ECD, not from LMICs, papers focusing on mHealth outcomes only and self-reporting interview or automated methods, giving us 36 relevant papers.

| **Technology** | **Purpose** | **Location** |
| --- | --- | --- |
| Cell phones [MESH]  Computers [MESH]  Handheld  Personal digital assistant  Tablet computer [MESH] | Surveys and questionnaires [MESH]  Data collection [MESH]  Data captur* | Developing countries [MESH]  Rural  Resource-poor  Health resource  Low resources |

**Web appendix 2 – Software used in LBWSAT**

**Software used in LBWSAT.**

FrontlineSMS (Occam Technologies - <http://www.frontlinesms.com>): This software was originally developed to enable simple data collection forms to be deployed on low-cost phones running the Symbian operating system (typically Nokia phones). Data collected with these forms would be sent to a server running FrontlineSMS software, using text messaging. The server software has general text message handling features, including the ability to have two-way communication with PHP (The PHP Group - <http://php.net>) scripts running on a web server. In this project we passed incoming text messages to a small PHP script that, amongst other things, used the source phone number of an incoming text message to determine the phone number of the appropriate interviewer and generated a message to the interviewer, which was passed back to FrontlineSMS for sending. We used FrontlineSMS 1.6, an old version that has some features not available in the current version.

Open Data Kit (ODK) (University of Washington, Seattle, WA - <https://opendatakit.org>): The heart of ODK is ODK Collect, a program to administer forms on Android mobile devices. Forms are designed as a specially formatted Excel workbook, which is then converted to an XForm .xml file, either locally (using software available at https://nafundi.com/blog/posts/xlsform-offline-easily-create-convert-and-validate-forms/) or using a web service (<http://opendatakit.org/xiframe/>). As well as ‘normal’ question types, ODK can capture images, audio, video and location. ODK forms can make use of external lookup tables, and can display images or other media as well as or instead of displaying text questions/answers. Form designers can use complicated logic to determine question routing and answer validity. Data collected with ODK can be transferred to a computer via USB cable or sent to an ODK Aggregate server. ODK Aggregate runs on a Tomcat (Apache Software Foundation - http://tomcat.apache.org) web server, either locally or in the cloud. We used ODK because it had all the functionality we needed for the Mobilisers and for the Endpoint survey, one of the team already had some experience of using ODK, and it was essentially free to use (we paid typically less than USD1 per month for the use of a Google AppSpot).

CommCare (Dimagi Inc. - <http://www.dimagi.com/products/>): This software is primarily targeted for use by health workers in developing countries, but it is also well suited to research data collection. The software for Android devices is based on ODK Collect, but it is also possible to use Java enabled phones for data collection. The main additional feature of CommCare over ODK is the ease with which it is possible to collect short-term longitudinal data (follow-ups) on study participants. This is done using so-called ‘case data’ that is stored on the mobile devices and is available during subsequent interactions. CommCare has an elaborate web interface, including a forms designer, form version control, user definition and access to data collected and submitted to the CommCare server. It is possible, but technically somewhat challenging, to set up the CommCare server software locally. Our main motivations for using CommCare were the ‘case data’ feature, and the availability of (both paid-for and free) support from Dimagi. We chose to use Dimagi’s server, rather than setting up our own. Using Dimagi’s server resulted in costs of around USD120 per month as we had more than the 50 users that can use the system without charge. We also used some features that are now only available to customers purchasing a subscription.

On the Android phones, we used a locking application called Hi App Lock (no longer available on the Google Play Store), to prevent phone users changing settings or running programs we didn’t want them to use. Because the users could then not switch their Internet connection on or off, we also used an application called Data Toggle Switch to restore that functionality (<https://play.google.com/store/apps/details?id=com.appsynapse.datatoggleadfree>).

Many other software products were used in our system. We used MySQL (Oracle Corporation - <http://www.mysql.com>) to store information about which Enumerators and Interviewers worked in which areas (and their phone numbers), and to log all SMS messages received from Enumerators and sent to Interviewers. Microsoft Access (Microsoft Corporation – <http://microsoft.com>) was used as a front end to this information. It was also used to produce the participant ID cards, and other reports. The QR codes on the ID cards were generated using the IDAutomation QR Code Active X control (IDAutomation.com Inc. - <http://www.idautomation.com/>). Stata SE 13.1 (StataCorp LP - <http://www.stata.com>) was used extensively to process the data collected, including as a scripting language to generate cURL (Daniel Stenberg - <http://curl.haxx.se>) commands to incrementally download data from servers.

Stata, Microsoft Access and the QR Code Active X control are commercial software. The other software used was free to use, and some open-source. Although CommCare is open-source, Dimagi, the developers, charged for our use of their server and services.
